# The Diverse Structures and Functions of Surfactant Proteins

**DOI:** 10.1016/j.tibs.2016.04.009

**Published:** 2016-07

**Authors:** Marieke Schor, Jack L. Reid, Cait E. MacPhee, Nicola R. Stanley-Wall

**Affiliations:** 1 School of Physics and Astronomy, University of Edinburgh, Edinburgh, UK; 2 School of Life Sciences, University of Dundee, Dundee, UK

## Abstract

Surface tension at liquid–air interfaces is a major barrier that needs to be surmounted by a wide range of organisms; surfactant and interfacially active proteins have evolved for this purpose. Although these proteins are essential for a variety of biological processes, our understanding of how they elicit their function has been limited. However, with the recent determination of high-resolution 3D structures of several examples, we have gained insight into the distinct shapes and mechanisms that have evolved to confer interfacial activity. It is now a matter of harnessing this information, and these systems, for biotechnological purposes.

## Trends

Interfacially active proteins fulfill a wide range of biological functions in organisms ranging from bacteria and fungi to mammals.

Their physicochemical properties make interfacially active proteins attractive for biotechnological applications; for example, as coatings on nanodevices or medical implants and as emulsifiers in food and personal-care products.

High-resolution 3D structures show that the mechanisms by which interfacially active proteins achieve their function are highly diverse.

## Life at the Air–Water Interface

**Figure 1:**
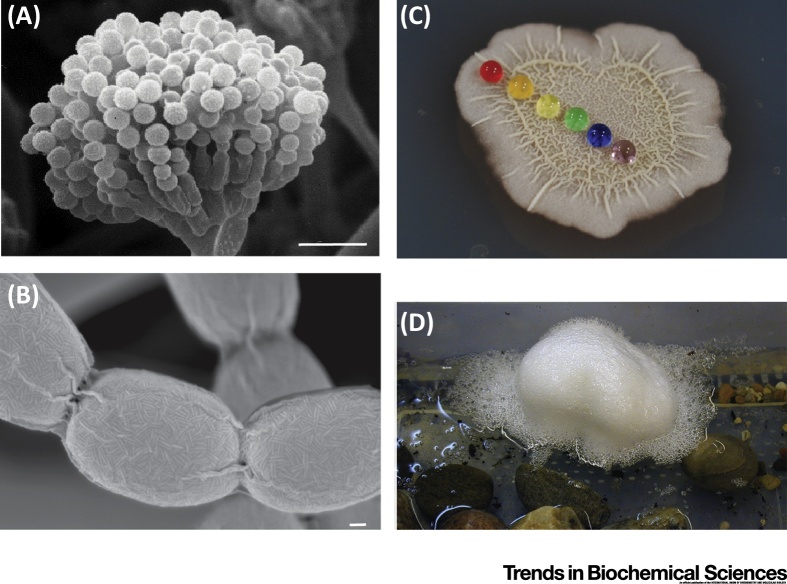
The Diverse Biological Functions of Interfacially Active Proteins. (A) Conidiophore development by *Aspergillus nidulans*. Image kindly provided by Professor Reinhard Fischer. Scale, 20 μm. (B) *Streptomyces coelicolor* rodlet formation on spores. Image kindly provided by Professor Marie Elliot and reprinted, with permission, from [39]. Scale, 100 nm. (C) *Bacillus subtilis* biofilm raincoat formation. Colored water droplets placed on a mature biofilm (Stanley-Wall laboratory). (D) A structured frog foam nest formed by *Engystomops pustulosus*. Image kindly provided by Dr Alan Cooper.

Overcoming surface tension at an air–water interface is crucial for many diverse biological processes, including sporulation of both bacteria and fungi, formation of foam nests during reproduction by frogs, and evaporative cooling in horses 1, 2, 3, 4, 5 (Table 1 and Figure 1). The desired reduction in surface tension is often mediated by **surfactant proteins** (see Glossary) (also known as surface-active or interfacially active proteins). This class of proteins can be roughly divided into three groups: those that function through associated lipids (which includes the pulmonary surfactants [6]), small **amphiphilic** peptides (e.g., surfactin of *Bacillus subtilis* [7]), and non-lipid-associated globular proteins, which are the focus of this review. Capitalizing on their biological activity, surfactant and surface-active proteins are expected to find numerous biotechnological applications; for example, as coatings of nanodevices or medical implants and as emulsifiers in food or personal-care products 8, 9.

In the past few years, the elucidation of high-resolution 3D structures of several surfactant proteins has opened the way for more detailed structure–function analysis of this exciting class of molecules 10, 11, 12, 13, 14, 15, 16, 17, 18. These studies reveal that the mechanisms by which surfactant proteins achieve their function are highly diverse. Some proteins are interfacially active in their native conformation, some refold, and others undergo (partial) restructuring. Furthermore, on association with the interface, some surface-active proteins remain monomeric while others organize into elastic films or other higher-order structures. Here we review recent progress in characterizing these structures, functions, and mechanisms and discuss how understanding the mode of protein engagement with the interface can inform potential biotechnological applications.

## Hydrophobins: Enabling Reproduction in Filamentous Fungi

Arguably the most comprehensively studied group of surfactant and surface-active proteins is the fungal hydrophobins. First discovered in *Schizophyllum commune* [19], they have subsequently been found to be ubiquitous among ascomycetes and basidiomycetes. These fungi typically live in moist environments and erect aerial **hyphae** to spread (Figure 1A) [20]. Under such conditions, surface tension at the air–water interface constitutes a significant barrier to hyphal erection. To overcome this barrier, the submerged hyphal cells secrete hydrophobins into the surrounding environment. The proteins assemble at the air–water interface, resulting in a significant decrease of the interfacial tension, which is essential for the hyphae to break through the surface 5, 21. Moreover, they form a hydrophobic coating on the aerial hyphae, fruiting bodies, and spores 9, 22. This coating not only allows the hyphae to colonize hydrophobic materials; it also facilitates dispersal of the spores into the air, promotes attachment to hydrophobic surfaces aiding invasion of hosts, and protects the spores of pathogenic fungi (e.g., *Aspergillus* sp.) from the host's immune system 22, 23.

All hydrophobins are small (7–9 kDa), moderately to highly hydrophobic proteins with low sequence similarity apart from eight conserved Cys residues that form four intramolecular disulfide bridges [9]. Traditionally, they have been divided into two classes based on the distribution of hydrophobic residues along the sequence and the higher-order structures they form on interaction with interfaces [21]. Class I hydrophobins have very little sequence similarity and on interaction with the interface undergo significant conformational changes to assemble into extremely stable fibrillar structures known as **rodlets** 5, 21, which share some structural similarity to amyloid-like fibrils [14]. Class II hydrophobins, by contrast, have somewhat higher sequence conservation and instead of assembling into rodlets these proteins form elastic monolayers 24, 25.

**Figure 2:**
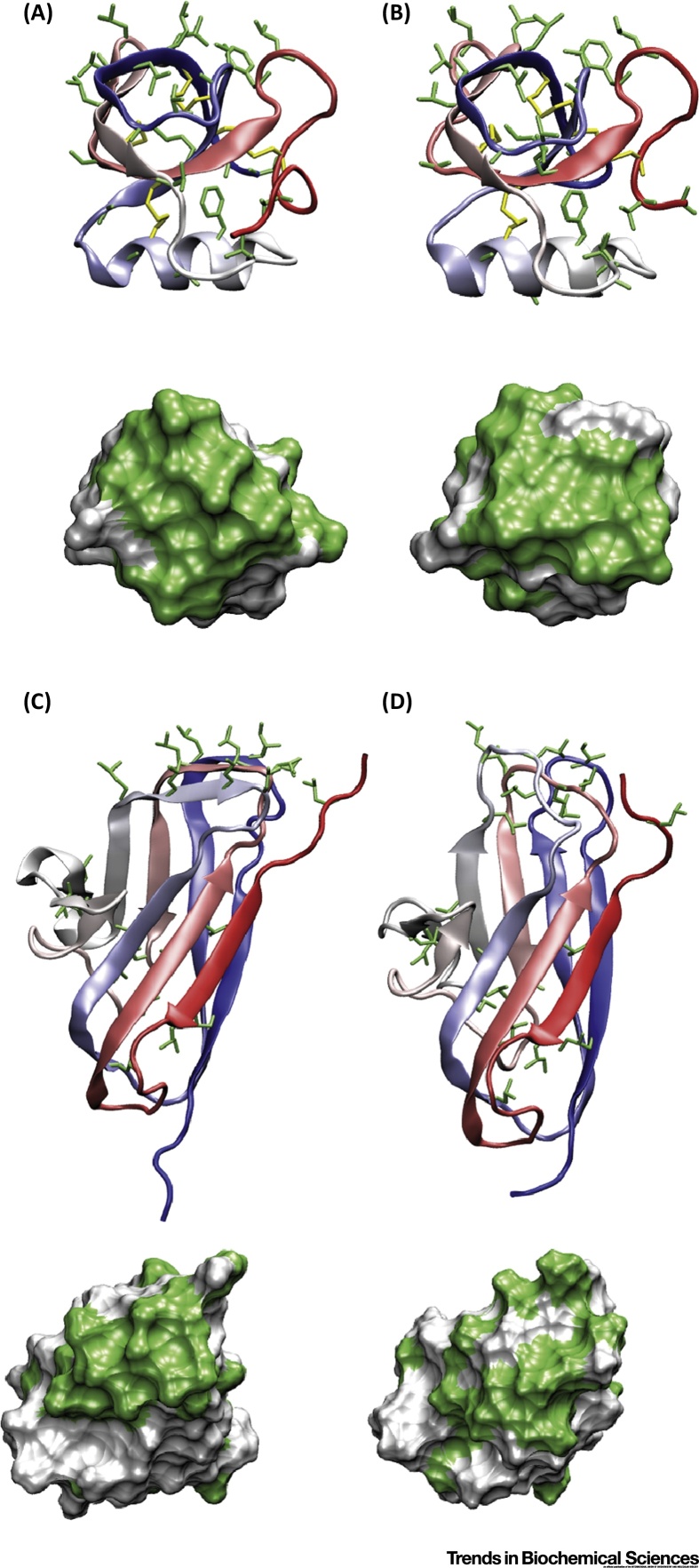
3D Structures of (A) Hydrophobin HFBI [11], (B) Hydrophobin HFBII [10], and Biofilm Surface Layer Protein A (BslA) in its Interfacially Active [13] (C), and Putative Water-Soluble (D) Forms 48, 49. Structures (top row) are shown side-on in cartoon representation and the color changes from red to blue from the N to the C terminus. The conserved disulfide bridges of HFBI and HFBII are highlighted in yellow and all hydrophobic amino acid side chains are highlighted in green. The bottom image shows the structures top-down in a surface representation with exposed hydrophobic areas shown in green and polar exposed surfaces in white. A clear exposed hydrophobic patch (measuring 783, 891, or 1620 Å^2^ for HFBI, HBII, and BslA, respectively) appended on a hydrophilic scaffold is seen for all three proteins. For both hydrophobins this patch is stabilized by the disulfide bridges. The BslA cap undergoes structural rearrangements to reduce the exposed hydrophobic surface (D). Images prepared using the Visual Molecular Dynamics package [86].

**Figure 3:**
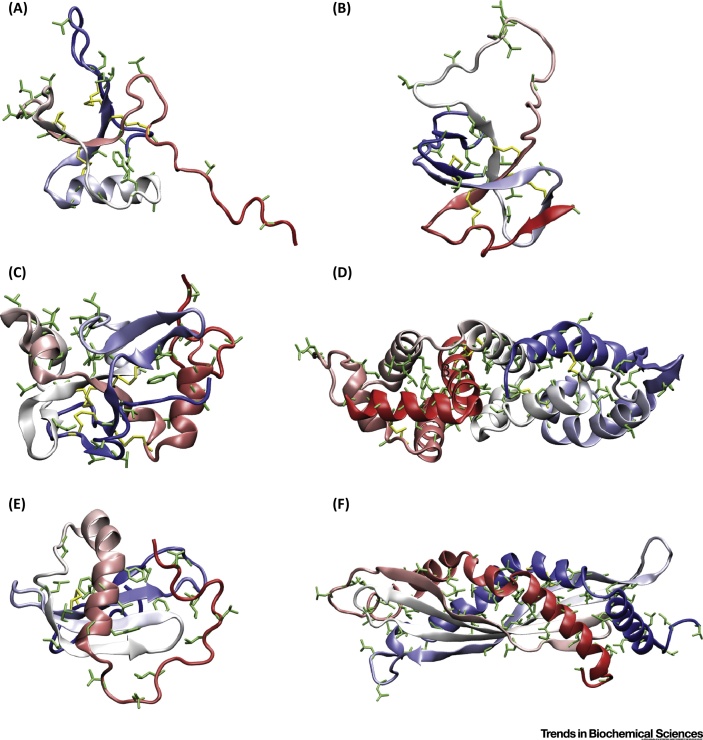
3D Structures of (A) NC2 [16], (B) EAS [14], (C) DewA [18], (D) Lv-Ranaspumin (Lv-Rsn) [12], (E) Rsn-2 [15], and (F) Latherin [17]. Structures are shown in cartoon representation and the color changes from red to blue from the N to the C terminus. All hydrophobic amino acid side chains are highlighted in green. Images prepared using the Visual Molecular Dynamics package [86]. (A–C) In these hydrophobins, some segregation of hydrophobic and hydrophilic residues can be seen although they have far more flexibility in their structures than HFBI, HFBII, and BslA. (D–F) Lv-Rsn, Rsn-2, and latherin are not obviously amphiphilic and most hydrophobic amino acid side chains are shielded from the solvent. However, these proteins are believed to undergo significant structural rearrangements on association with an air–water interface.

The determination of several high-resolution structures of both class I (EAS of *Neurospora crassa* [14] and DewA of *Aspergillus nidulans* [18]) and class II (HFBI and HFBII from *Trichoderma reesei* 10, 11 and NC2 from *N. crassa* [16]) hydrophobins has added significant understanding to how these proteins mediate their biological function (Figure 2A,B and Figure 3A,B,C). Structures of both classes share a β sheet core. In the three class II hydrophobins, this sheet is rolled up to form a β barrel, which is held together by intramolecular hydrogen bonds. A single α helix and two relatively ordered loops are tethered to the β barrel via two of the four conserved disulfide bridges, resulting in a structure with limited flexibility [10]. The two class I hydrophobins, by contrast, have two flexible loops appended to an open β sheet 14, 18. Both EAS and DewA have putative aggregation-prone (amyloidogenic) regions in one of their loops. Once the EAS protein associates with the air–water interface, the loop containing the amyloidogenic sequence changes conformation to form an intermolecularly hydrogen-bonded cross-β core [26]. Although the location and sequence of the amyloidogenic region is not conserved in DewA, it is likely that a similar mechanism is involved [18].

Importantly, all five structures are amphiphilic, meaning that there is clear segregation of hydrophobic and polar residues on the outside of the proteins (Figure 2A,B and Figure 3A,B,C), thus explaining their affinity for the air–water interface. In HFBI and HFBII, the large hydrophobic patch is held in a surface-exposed orientation by the disulfide bridge scaffold. In aqueous solution, oligomerization into dimers or tetramers shields it from solvent [11]. By contrast, NC2, EAS, and DewA are (largely) monomeric in aqueous solution 9, 14, 18. This is likely to be mediated by the more polar, disordered regions of these proteins [27]; that is, the loops of EAS and DewA and the N terminus of NC2 are thought to shield the hydrophobic patches from the solvent and obstruct self-assembly. When adsorbed to the interface, the orientation of these proteins is constrained, facilitating further assembly either into rodlets as discussed above or into films in case of the class II hydrophobins 28, 29. Within these elastic films, the class II hydrophobins form specific intermolecular hydrophobic or charged interactions, thereby enhancing film stability 16, 30, 31, 32. The substantial free energy of adsorption to the interface, which has been estimated to be around 100*k*_B_*T* for these proteins [33], ensures that the hydrophobins monomerize to interact with the interface through their hydrophobic patch.

## Same Function, Different Set of Proteins

The life cycle of filamentous bacteria has similarities to that of filamentous fungi, including the projection of aerial mycelia from submerged substrate mycelia to allow sporulation and subsequent spore dispersal (Figure 1B). Consistent with this, the best-studied filamentous bacterium, *Streptomyces coelicolor*, produces two groups of interfacially active proteins (rodlins 34, 35 and chaplins 1, 2) (Table 1) that combine forces with a ‘lantibiotic-like’ lanthionine-containing peptide, SapB 36, 37, 38, to facilitate the developmental life cycle. SapB and two of the chaplins, ChpE and ChpH, are excreted by the submerged mycelium and their main role is in lowering the surface tension 38, 39. The other chaplins are predominantly expressed after hyphae have been erected and, together with ChpE and ChpH, assemble to form a hydrophobic spore coating, although they also help to further reduce the surface tension. This coating is further organized into rodlets, similar to those observed for class I hydrophobins, likely in combination with the rodlin proteins (RdlA and/or RdlB) although biochemical or cell biology data to support this are currently lacking. The rodlin proteins are particularly important under conditions of high osmolarity, where lack of rodlin production blocks *S. coelicolor* aerial hyphae formation [40].

So far, no high-resolution structural information is available for any of the *S. coelicolor* surface-active proteins. It should be noted that all of the chaplins have significant interfacial activity in isolation [41]. Circular dichroism measurements indicate that the chaplins, which comprise one (short chaplins, ChpD–H) or two (long chaplins, ChpA–C) chaplin domains [39], are either predominantly disordered in solution or adopt a mixture of random coil and β sheet secondary structure [41]. It has been proposed that formation of the amyloid-like chaplin fibrils, which constitute the rodlet layer together with the rodlin proteins 1, 34, occurs by a two-step process [42]. Monomeric chaplins are predicted to transition through a semiliquid membrane state that exhibits high surface activity, and where additional monomers can still be inserted, before adopting a more rigid β sheet conformation that covers the hyphae [42]. High-resolution structural data will be critical for further understanding of the mechanism of this process.

## A Biofilm Raincoat Formed by Biofilm Surface Layer Protein A (BslA)

Interfacially active proteins also have biological functions in non-filamentous microorganisms. The best-studied example is the *B. subtilis* protein BslA 13, 43 (Table 1). *B. subtilis* is a Gram-positive, soil-dwelling bacterium that can live in association with plant roots. It has an ability to induce systemic resistance in plants, thus conferring protection against pathogenic bacteria and fungi. This process is dependent on both its capacity to form a surface-associated bacterial aggregate called a **biofilm** [44] and its capability to produce a wide range of bioactive molecules, including antibiotics [45]. When resident within a biofilm, the bacterial cells are surrounded by a self-produced extracellular polymer matrix mainly comprising exopolysaccharides and protein fibers [46]. In the *B. subtilis* biofilm, BslA forms a hydrophobic layer on the outside of this matrix, rendering the mature assembly virtually impenetrable to hydrophilic molecules 43, 47 (Figure 1C). The striking nature of the protection conferred has led to BslA being referred to as a bacterial ‘raincoat’.

X-ray crystallography revealed that, at a structural level, BslA comprises two long β sheets, one four stranded and one three stranded, that stack together [13] (Figure 2C). Appended onto this scaffold is a small loop comprising a 3^10^ helix and three short β strands. Despite low sequence similarity, the fold identifies BslA as a member of the Ig superfamily. It can be argued that the most striking feature of the BslA structure is the small, three-stranded β sheet that is positioned above the Ig fold like a ‘cap’. For eight of the monomers in the decameric crystal structure, there is an unusually high number of solvent-exposed hydrophobic amino acids in this region. These exposed hydrophobic residues are protected from the solvent by crystal contacts with the other proteins in the asymmetric unit, which resembles a micelle with the cap regions of each monomer oriented towards the dry/solvent-free interior of the decamer.

Although structurally very different, a feature BslA shares with the fungal hydrophobins is that it is obviously amphiphilic, with the hydrophilic Ig domain favoring the solvent and the hydrophobic cap favoring the air. Such large solvent-exposed hydrophobic patches are inherently unstable and the hydrophobins have very rigid structures, with four disulfide bridges that keep the hydrophobic patch in place (as discussed above) (Figure 2A,B). BslA, however, lacks these stabilizing disulfide bridges, and although it crystallizes as a decamer it is stable in a monomeric form in solution [48]. Circular dichroism reveals that BslA undergoes a limited conformational change on association with the air–water interface. In solution the structure is more disordered due to reorientation of the hydrophobic amino acids in the cap region towards the interior of the protein (Figure 2D) so that the hydrophobic cap is deployed only in the right place and at the right time 48, 49.

On insertion into the interface, BslA forms an elastic film similar to those observed for class II hydrophobins 13, 48, 50. The speed and strength of adsorption and the stability of the film can be tuned by mutating the exposed hydrophobic residues in the cap 13, 49. Reducing the overall hydrophobicity of the cap region reduces the attraction between this domain and the air–oil phase. This affects how BslA inserts into the interface: the Pickering energy, which favors orientations where the long axis of the protein aligns with the interface, starts to dominate the free energy of insertion [49]. This in turn disrupts the lateral interactions that stabilize the BslA film, leading to partial or even complete loss of the raincoat function in the biofilm [13].

## Stabilizing Frog Foam Nests with Ranaspumins

The strategy of lowering of the surface tension through the production of interfacially active proteins and peptides is not restricted to microorganisms but has also been observed in the animal kingdom, for instance in frog foam nests. Some species of tropical and subtropical frogs produce structured foam nests to protect fertilized eggs and tadpoles from dehydration and microbial infection [51] (Figure 1D). Foams comprise small air bubbles separated by liquid and foam formation is facilitated by reducing the surface tension of the air–liquid interface combined with vigorous mixing. The resulting air-in-water emulsions are relatively unstable as the air bubbles will coalesce or burst over time, leading to eventual collapse of the foam [52]. However, frog foam nests are stable for multiple days, or even weeks, under harsh tropical conditions due to the addition of surfactant species. That said, foams generated *in vitro* using isolated interfacially active protein components are unstable, indicating that the native nest foams must contain additional stabilizing factors [8].

Analysis of the foam nests of the tropical frog *Engystomops pustulosus* revealed six unknown proteins, subsequently called ranaspumins (RSN1–6) 3, 51 (Table 1). Ep-RSN-2 is the major surfactant protein in the mix, with the other ranaspumins contributing to foam stability and defense against microbes. Similarly, analysis of the foam nests of an unrelated frog species, *Leptodactylus vastus*, revealed a mix of proteins with Lv-ranaspumin (Lv-RSN-1) being the main contributor to the interfacial activity of the mixture 12, 53 (Table 1).

High-resolution structural analysis of Ep-Rsn-2 and Lv-Rsn-1 by NMR and X-ray crystallography, respectively, revealed no structural similarities between the proteins (Figure 3D,E) 12, 15. Lv-Rsn-1 is a 23.5-kDa protein comprising two domains [12]. The N-terminal domain comprises a bundle of six antiparallel α helices. The C-terminal domain contains a sheet of three antiparallel α helices and two short β strands with a fourth helix lying across the sheet. The structure is stabilized by four disulfide bridges, although this similarity to the fungal hydrophobins is coincidental and the proteins share neither sequence nor structural homology. By contrast, Ep-Rsn-2, which is only 11 kDa in size, comprises a four-stranded antiparallel β sheet with a slightly kinked α helix lying across one side of the sheet [15]. This fold is characteristic of cystatins; however, Ep-Rsn-2 shows no protease activity [15].

Both Ep-Rsn-2 and Lv-Rsn-1 are monomeric in solution and, in contrast to the classes of proteins described earlier, neither of these structures is obviously **amphipathic**. It has therefore been postulated that both proteins need to undergo some conformational change to facilitate interfacial association. Two possible conformational changes have been suggested for Lv-Rsn-1: the β strands could move to increase the hydrophobic cavity or the two halves of the protein could move apart to expose the hydrophobic core [12]. For Ep-Rsn-2, clamshell-like opening of the protein in which the helix unhinges from the β sheet has been hypothesized and is consistent with neutron-scattering data [15]. This process has been tested by coarse-grained simulations, supported by experimental data, that indicate that ep-Rsn-2 adsorption to interfaces is a two-step process [54]. First, the flexible N-terminal tail captures the interface. This is followed by a large conformational change where the helix ‘unhinges’ from the β sheet revealing the hydrophobic core. The protein inserts into the interface but all secondary structural elements are retained.

## Latherin: The Controlling Factor behind Foaming Horse Sweat

The final example of an evolved surfactant protein discussed here is latherin (Table 1), which is found in the sweat and saliva of horses and other equines [55]. Like humans, during exercise horses regulate their body temperature by sweating. However, while human sweat has a high salt concentration and little protein, horse sweat has the converse composition: it is low in salt and contains a high concentration of protein. For evaporative cooling to work, the sweat has to rapidly make its way from the sweat glands in the dermis to the air interface. However, as horses have thick pelts of oily, waterproof hair, this is not as straightforward as in humans. The surfactant properties of latherin, the main protein component of horse sweat, enable wetting of the hairs thereby aiding fast flow of the sweat through the pelt [4]. Latherin is also present in horse saliva, where it has been postulated to aid mastication and penetration of digestive enzymes into the dry, fibrous food consumed by equines [4].

The amino acid sequences of latherin from different equine species are highly conserved. Latherins belong to the group of the palate, lung, and nasal epithelium clone (PLUNC) proteins expressed in mammalian salivary glands and oral cavities 4, 56. The biological roles of the PLUNC proteins are not well understood, although there is some evidence that they are involved in host defense [56] and at least some of the PLUNC proteins have significant interfacial activity [57].

A high-resolution structure of latherin in solution has been determined using NMR (Figure 3F) [17]. Latherin is monomeric in solution and comprises a long, four-stranded β sheet onto which two antiparallel α helices are packed, giving the structure an almost cylindrical shape with two flexible loops sticking out from one end. Analogous to the ranaspumins, the solution structure of latherin displays no obvious amphiphilicity, with most hydrophobic amino acids evenly distributed along the length of the structure and confined to the interior. A mechanism involving major conformational changes has been proposed for interfacial association of latherin [17]. Initial recognition of the interface is thought to occur through the flexible loops, which have a relatively high leucine content. This is proposed to involve unzipping of the two helices resulting in an open, planar conformation in which the individual secondary structure elements are retained and the full hydrophobic core is exposed to the interface. The dimensions of the planar, unfolded protein are in reasonable agreement with neutron-reflection measurements, which indicated that latherin forms a thin monolayer at the interface 4, 17.

## Potential Applications of Interfacially Active Proteins

The physicochemical characteristics that allow surfactant and surface-active proteins to perform their biological roles are also sought after for many biotechnological applications 8, 9, 58. These proteins can associate with hydrophobic or hydrophilic surfaces, thereby inverting the character of the surface [42]. Rendering hydrophobic surfaces hydrophilic is of particular interest as this greatly enhances cell attachment to and growth on these surfaces. Conversely, rendering a surface hydrophobic can, for example, prevent biofouling, which is the undesired growth of microorganisms on surfaces such as pipes and catheters. Further to this, the self-assembly process of hydrophobins is very robust and functional groups can be added to the proteins allowing selective capturing of cell types. Thus, these proteins could, for instance, be used to enhance the biocompatibility of medical implants [59]. Moreover, hydrophobins can be deposited to create patterns that will be maintained due to the difference in wettability between the coated and uncoated surfaces [60]. This is of particular interest for applications in tissue engineering.

Interfacially active proteins can also be used to enhance the solubility of poorly water-soluble drugs [61]. Self-assembling interfacially active proteins, like the hydrophobins and potentially BslA, can form cages around these hydrophobic drug molecules or can be used to increase the solubility and biocompatibility of drug carriers such as silicon nanoparticles and carbon nanotubes 62, 63, 64. Expansion in this area may increase the range of molecules that can be used for future drug development.

The amphiphilic character of interfacially active proteins can also be applied to enhance the formation and stability of foams and emulsions, which is of particular interest for the food and personal-care industries 8, 65, 66 but is also applicable to any multiphase formulation. A common problem in this type of product when it is stabilized by traditional surfactants is the coarsening of the structure due to coalescence of the oil or air droplets over time. The thin, solid, stable elastic film formed by BslA or the hydrophobins around these droplets inhibits this process, contributing significantly to the stability of products such as ice cream 66, 67.

While protein film formation is clearly beneficial in the applications discussed above, it is not always desirable. Non-film-forming interfacially active peptides, like the ranaspumins and latherin, are attractive candidates for applications where biocompatibility and increased wettability are desired but the formation of very long-lived stable foams is not; for example, in agricultural sprays [68] or as spray-on foams to provide vital short-term protection for complicated wounds such as those of burn victims [8]. In combination, these proteins, with their distinct suite of mechanisms to impart interfacial activity, provide opportunities for exploitation in a wide range of potential applications.

## Concluding Remarks

Interfacially active proteins fulfill a wide range of biological functions in organisms ranging from bacteria and fungi to amphibians and mammals. The recent elucidation of high-resolution structures of several of these proteins has made it clear that their functional diversity is underpinned by both structural and protein sequence diversity. The high-resolution structures highlight that divergent and convergent mechanisms have evolved to enable interfacially active proteins to perform their specific biological functions; for example, at biofilm–air, aerial hyphae–air, and horse pelt–air interfaces (Figure 2, Figure 3). Both the ranaspumins and latherin undergo major conformational rearrangements, essentially opening the hydrophobic core but avoiding complete denaturation, to stably associate with the surface. By contrast, the hydrophobins are already obviously amphipathic and have rigid structures held together by a network of disulfide bridges. BslA can be viewed as an intermediate case in that it undergoes only a limited, localized conformational change to enhance association with an interface. On interaction with the surface, type I hydrophobins and chaplins form rodlets, type II hydrophobins and BslA form elastic films, and the ranaspumins and latherins seem to form monolayers of non-interacting proteins. Ongoing efforts to characterize these higher-order assemblies, particularly using solid-state NMR, will hopefully shed light on these modes of action.

Continuing efforts to identify new interfacially active proteins and elucidate their structures and functional mechanisms will hopefully allow the development of systematic screens for this fascinating class of proteins (see Outstanding Questions). Improving our understanding of their structure–function relationships, as well as the assemblies they form at interfaces, is a crucial step towards the rational design and optimization of these proteins for the food, personal-care, and medical industries.

Outstanding Questions How many distinct mechanisms have evolved to impart interfacial activity? The recent elucidation of several high-resolution structures of interfacially active proteins highlights that these are various ways to satisfy the seemingly contradictory criteria of having a stable, water-soluble and yet surface-active protein. However, it is unclear how common these mechanisms are and there may be many more, as-yet-undiscovered mechanisms.

Can systematic approaches be developed to identify new interfacially active proteins? To date, interfacially active proteins have been discovered by serendipity and it seems likely that there are many more in nature. A systematic and preferably bioinformatics-based method to search for these versatile proteins would greatly enhance this field of study.

How can interfacially active proteins be optimized for use in biotechnological applications? New insights into the 3D structures and functional mechanisms of interfacially active proteins as well as an improved understanding of their higher-order assemblies at interfaces opens ways to design, engineer, and fine-tune their behavior to suit various applications; for example, in food, cosmetics, and medical implants.

## Figures and Tables

**Table 1:** Interfacially Active Proteins and Their Biological Functions

Protein	Species	*In vivo* Function	PDB ID^a^	*In vitro* Structure^b^	Surface Tension Reduction (from 73 mN/m)^c^	Refs
BslA	*Bacillus subtilis*	Hydrophobic coating of biofilms	4BHU (crystal)	2D lattice film (TEM)	−	13, 48
Chaplins (ChpA–H)	*Streptomyces coelicolor*	Projection of aerial hyphae, surface attachment	−	Rodlets (shadowing, SEM)	26	1, 69
Rodlins	*Streptomyces* sp.	Hydrophobic coating of hyphae	−	Rodlets (SEM)	−	[35]
ABH1	*Agaricus bisporus*	Hydrophobic coating of fruiting bodies and lining air channels	−	Rodlets (EM)	−	70, 71, 72
ABH3	*A. bisporus*	Lowering surface tension to enable projection of aerial hyphae	−	Rodlets (shadowing)	37	[73]
HYD1/2	*Beauveria bassiana*	Hydrophobic spore coating, cell surface adhesion	−	Rodlets (AFM, SEM)	−	[74]
SC3	*Schizophyllum commune*	Lowering surface tension to enable projection of aerial hyphae and attachment to hydrophobic surfaces	−	Rodlets (EM)	32	5, 75, 76
SC4	*S. commune*	Hydrophobic lining of fruiting-body gas channels	−	Rodlets (shadowing, EM)	36	72, 75
RodA	*Aspergillus* sp.	Hydrophobic spore coating	−	Rodlets (TEM, SEM)	−	77, 78, 79
DewA	*Aspergillus nidulans*	Spore hydrophobicity	2LSH (NMR)	Rodlets (TEM)	−	18, 79
EAS	*Neurospora crassa*	Hydrophobic spore coating	2FMC (NMR)	Rodlets (AFM)	−	14, 80
MPG1	*Magnaporthe grisea*	Hydrophobic spore coating, surface adhesion	−	Rodlets (TEM)	−	81, 82
HFBI	*Trichoderma reesei*	Projection of aerial hyphae	2FZ6 (crystal)	Monolayers	25	11, 83, 84
HFBII	*T. reesei*	Projection of aerial hyphae	1R2M (crystal)	Monolayers	25	10, 83, 85
NC2	*N. crassa*	Unclear	4AOG (NMR)	Monolayers (AFM)	−	[16]
Rsn-2	*Engystomus pustulus*	Reduces surface tension enabling foam nest formation	2WGO (NMR)	Monolayers (IR, neutron reflectivity)	52	[15]
Lv-Rsn-1	*Leptodactylus vastus*	Reduces surface tension enabling foam nest formation	4K83 (crystal)	Unknown	61	[12]
Latherin	*Equus ferus*	Wetting of pelt (sweating) and food (mastication)	3ZPM (NMR)	Dense layer (neutron reflectivity)	56	4, 17, 55

a Crystal, X-ray crystallography; NMR, nuclear magnetic resonance spectroscopy.

b TEM, transmission electron microscopy; SEM, scanning electron microscopy; AFM, atomic force microscopy; IR, infrared spectroscopy.

c These values are provided as a guide to the interfacial activity and are typically calculated by estimating the surface tension from the droplet/bubble shape. When comparing proteins it is important to note that, as protein films or rodlets form, the measurement is no longer valid as the droplet deforms.

## Glossary

Amphipathic/amphiphilic: a molecule with both hydrophilic and hydrophobic characteristics. When applied to globular proteins, the term refers to distinct hydrophilic and hydrophobic surface regions.
Biofilm: a community of microbial cells encased in a self-produced extracellular matrix comprising a mixture of proteins, carbohydrate, extracellular DNA, and frequently lipids.
Hyphae: long, filamentous growth form of a fungus or filamentous bacterium. These comprise the mycelia.
Rodlet: a self-assembling, proteinaceous, rod-shaped structure that is shorter and typically has a higher persistence length than a protein fibril or filament.
Surfactant proteins: proteins that decrease the interfacial tension at an air–water or liquid–liquid interface. We use the terms ‘surfactant’, ‘surface active’, and ‘interfacially active’ interchangeably in this review; however, it should be noted that some proteins are interfacially active without decreasing the apparent interfacial tension. This is because some proteins form a thin elastic film at the interface and the concept of ‘interfacial tension’ no longer applies.

## References

[1] Claessen D. (2003). A novel class of secreted hydrophobic proteins is involved in aerial hyphae formation in *Streptomyces coelicolor* by forming amyloid-like fibrils. Genes Dev..

[2] Elliot M.A. (2003). The chaplins: a family of hydrophobic cell-surface proteins involved in aerial mycelium formation in *Streptomyces coelicolor*. Genes Dev..

[3] Fleming R.I. (2009). Foam nest components of the tungara frog: a cocktail of proteins conferring physical and biological resilience. Proc. Biol. Sci..

[4] McDonald R.E. (2009). Latherin: a surfactant protein of horse sweat and saliva. PLoS ONE.

[5] Wosten H.A.B. (1993). Interfacial self-assembly of a fungal hydrophobin into a hydrophobic rodlet layer. Plant Cell.

[6] Whitsett J.A., Weaver T.E. (2002). Hydrophobic surfactant proteins in lung function and disease. N. Engl. J. Med..

[7] Nakano M.M. (1992). Isolation and characterization of *sfp*: a gene that functions in the production of the lipopeptide biosurfactant, surfactin, in *Bacillus subtilis*. Mol. Gen. Genet..

[8] Cooper A., Kennedy M.W. (2010). Biofoams and natural protein surfactants. Biophys. Chem..

[9] Ren Q. (2013). Two forms and two faces, multiple states and multiple uses: properties and applications of the self-assembling fungal hydrophobins. Biopolymers.

[10] Hakanpaa J. (2004). Atomic resolution structure of the HFBII hydrophobin, a self-assembling amphiphile. J. Biol. Chem..

[11] Hakanpaa J. (2006). Two crystal structures of *Trichoderma reesei* hydrophobin HFBI–the structure of a protein amphiphile with and without detergent interaction. Protein Sci..

[12] Hissa D.C. (2014). Unique crystal structure of a novel surfactant protein from the foam nest of the frog *Leptodactylus vastus*. Chembiochem.

[13] Hobley L. (2013). BslA is a self-assembling bacterial hydrophobin that coats the *Bacillus subtilis* biofilm. Proc. Natl. Acad. Sci. U.S.A..

[14] Kwan A.H.Y. (2006). Structural basis for rodlet assembly in fungal hydrophobins. Proc. Natl. Acad. Sci. U.S.A..

[15] Mackenzie C.D. (2009). Ranaspumin-2: structure and function of a surfactant protein from the foam nests of a tropical frog. Biophys. J..

[16] Ren Q. (2014). Solution structure and interface-driven self-assembly of NC2, a new member of the class II hydrophobin proteins. Proteins.

[17] Vance S.J. (2013). The structure of latherin, a surfactant allergen protein from horse sweat and saliva. J. R. Soc. Interface.

[18] Morris V.K. (2013). Analysis of the structure and conformational states of DewA gives insight into the assembly of the fungal hydrophobins. J. Mol. Biol..

[19] Schuren F.H., Wessels J.G. (1990). Two genes specifically expressed in fruiting dikaryons of *Schizophyllum commune*: homologies with a gene not regulated by mating-type genes. Gene.

[20] Wosten H.A. (1999). How a fungus escapes the water to grow into the air. Curr. Biol..

[21] Wosten H.A. (2001). Hydrophobins: multipurpose proteins. Annu. Rev. Microbiol..

[22] Bayry J. (2012). Hydrophobins–unique fungal proteins. PLoS Pathog..

[23] Aimanianda V. (2009). Surface hydrophobin prevents immune recognition of airborne fungal spores. Nature.

[24] Paananen A. (2003). Structural hierarchy in molecular films of two class II hydrophobins. Biochemistry.

[25] Torkkeli M. (2002). Aggregation and self-assembly of hydrophobins from *Trichoderma reesei*: low-resolution structural models. Biophys. J..

[26] Macindoe I. (2012). Self-assembly of functional, amphipathic amyloid monolayers by the fungal hydrophobin EAS. Proc. Natl. Acad. Sci. U.S.A..

[27] Abeln S., Frenkel D. (2008). Disordered flanks prevent peptide aggregation. PLoS Comput. Biol..

[28] Kisko K. (2009). Self-assembled films of hydrophobin proteins HFBI and HFBII studied *in situ* at the air/water interface. Langmuir.

[29] Morris V.K. (2011). Recruitment of class I hydrophobins to the air:water interface initiates a multi-step process of functional amyloid formation. J. Biol. Chem..

[30] Basheva E.S. (2011). Self-assembled bilayers from the protein HFBII hydrophobin: nature of the adhesion energy. Langmuir.

[31] Lienemann M. (2015). Charge-based engineering of hydrophobin HFBI: effect on interfacial assembly and interactions. Biomacromolecules.

[32] Magarkar A. (2014). Hydrophobin film structure for HFBI and HFBII and mechanism for accelerated film formation. PLoS Comput. Biol..

[33] Cheung D.L. (2012). Molecular simulation of hydrophobin adsorption at an oil–water interface. Langmuir.

[34] Claessen D. (2004). The formation of the rodlet layer of streptomycetes is the result of the interplay between rodlins and chaplins. Mol. Microbiol..

[35] Claessen D. (2002). Two novel homologous proteins of *Streptomyces coelicolor* and *Streptomyces lividans* are involved in the formation of the rodlet layer and mediate attachment to a hydrophobic surface. Mol. Microbiol..

[36] Kodani S. (2004). The SapB morphogen is a lantibiotic-like peptide derived from the product of the developmental gene *ramS* in *Streptomyces coelicolor*. Proc. Natl. Acad. Sci. U.S.A..

[37] Tillotson R.D. (1998). A surface active protein involved in aerial hyphae formation in the filamentous fungus *Schizophillum commune* restores the capacity of a bald mutant of the filamentous bacterium *Streptomyces coelicolor* to erect aerial structures. Mol. Microbiol..

[38] Willey J. (1991). Extracellular complementation of a developmental mutation implicates a small sporulation protein in aerial mycelium formation by *S. coelicolor*. Cell.

[39] Di Berardo C. (2008). Function and redundancy of the chaplin cell surface proteins in aerial hypha formation, rodlet assembly, and viability in *Streptomyces coelicolor*. J. Bacteriol..

[40] de Jong W. (2012). SapB and the rodlins are required for development of *Streptomyces coelicolor* in high osmolarity media. FEMS Microbiol. Lett..

[41] Sawyer E.B. (2011). The assembly of individual chaplin peptides from *Streptomyces coelicolor* into functional amyloid fibrils. PLoS ONE.

[42] Ekkers D.M. (2014). Surface modification using interfacial assembly of the *Streptomyces* chaplin proteins. Appl. Microbiol. Biotechnol..

[43] Kobayashi K., Iwano M. (2012). BslA (YuaB) forms a hydrophobic layer on the surface of *Bacillus subtilis* biofilms. Mol. Microbiol..

[44] Morikawa M. (2006). Beneficial biofilm formation by industrial bacteria *Bacillus subtilis* and related species. J. Biosci. Bioeng..

[45] Bais H.P. (2004). Biocontrol of *Bacillus subtilis* against infection of *Arabidopsis* roots by *Pseudomonas syringae* is facilitated by biofilm formation and surfactin production. Plant Physiol..

[46] Hobley L. (2015). Giving structure to the biofilm matrix: an overview of individual strategies and emerging common themes. FEMS Microbiol. Rev..

[47] Ostrowski A. (2011). YuaB functions synergistically with the exopolysaccharide and TasA amyloid fibers to allow biofilm formation by *Bacillus subtilis*. J. Bacteriol..

[48] Bromley K.M. (2015). Interfacial self-assembly of a bacterial hydrophobin. Proc. Natl Acad. Sci. U.S.A..

[49] Brandani G.B. (2015). The bacterial hydrophobin BslA is a switchable ellipsoidal Janus nanocolloid. Langmuir.

[50] Wang Z.G. (2016). A narrow amide I vibrational band observed by sum frequency generation spectroscopy reveals highly ordered structures of a biofilm protein at the air/water interface. Chem. Commun. (Camb.).

[51] Cooper A. (2005). Adsorption of frog foam nest proteins at the air–water interface. Biophys. J..

[52] Pugh R.J. (1996). Foaming, foam films, antifoaming and defoaming. Adv. Colloid Interface Sci..

[53] Hissa D.C. (2008). Novel surfactant proteins are involved in the structure and stability of foam nests from the frog *Leptodactylus vastus*. J. Exp. Biol..

[54] Morris R.J. (2016). The conformation of interfacially adsorbed ranaspumin-2 is an arrested state on the unfolding pathway. arXiv.

[55] Beeley J.G. (1986). Isolation and characterization of latherin, a surface-active protein from horse sweat. Biochem. J..

[56] Bingle C.D., Craven C.J. (2002). PLUNC: a novel family of candidate host defence proteins expressed in the upper airways and nasopharynx. Hum. Mol. Genet..

[57] Gakhar L. (2010). PLUNC is a novel airway surfactant protein with anti-biofilm activity. PLoS ONE.

[58] Wosten H.A., Scholtmeijer K. (2015). Applications of hydrophobins: current state and perspectives. Appl. Microbiol. Biotechnol..

[59] Boeuf S. (2012). Engineering hydrophobin DewA to generate surfaces that enhance adhesion of human but not bacterial cells. Acta Biomater..

[60] Li X.X. (2009). Patterning of neural stem cells on poly(lactic-co-glycolic acid) film modified by hydrophobin. Colloids Surf. B Biointerfaces.

[61] Valo H.K. (2010). Multifunctional hydrophobin: toward functional coatings for drug nanoparticles. ACS Nano.

[62] Wang X.S. (2010). Noncovalently functionalized multi-wall carbon nanotubes in aqueous solution using the hydrophobin HFBI and their electroanalytical application. Biosens. Bioelectron..

[63] Yang W.R. (2013). Surface functionalization of carbon nanomaterials by self-assembling hydrophobin proteins. Biopolymers.

[64] Haas Jimoh Akanbi M. (2010). Use of hydrophobins in formulation of water insoluble drugs for oral administration. Colloids Surf. B Biointerfaces.

[65] Dickinson E. (2015). Colloids in food: ingredients, structure, and stability. Annu. Rev. Food. Sci. Technol..

[66] Green A.J. (2013). Formation and stability of food foams and aerated emulsions: hydrophobins as novel functional ingredients. Curr. Opin. Colloid Interface Sci..

[67] Stanley-Wall N.R., MacPhee C.E. (2015). Connecting the dots between bacterial biofilms and ice cream. Phys. Biol..

[68] Frey S.L. (2015). A non-foaming proteosurfactant engineered from ranaspumin-2. Colloids Surf. B Biointerfaces.

[69] Capstick D.S. (2007). SapB and the chaplins: connections between morphogenetic proteins in *Streptomyces coelicolor*. Mol. Microbiol..

[70] DeGroot P.W.J. (1996). The *Agaricus bisporus hypA* gene encodes a hydrophobin and specifically accumulates in peel tissue of mushroom caps during fruit body development. J. Mol. Biol..

[71] Lugones L.G. (1996). An abundant hydrophobin (ABH1) forms hydrophobic rodlet layers in *Agaricus bisporus* fruiting bodies. Microbiology.

[72] Lugones L.G. (1999). Hydrophobins line air channels in fruiting bodies of *Schizophyllum commune* and *Agaricus bisporus*. Mycol. Res..

[73] Lugones L.G. (1998). A hydrophobin (ABH3) specifically secreted by vegetatively growing hyphae of *Agaricus bisporus* (common white button mushroom). Microbiology.

[74] Zhang S.Z. (2011). Two hydrophobins are involved in fungal spore coat rodlet layer assembly and each play distinct roles in surface interactions, development and pathogenesis in the entomopathogenic fungus, *Beauveria bassiana*. Mol. Microbiol..

[75] van Wetter M.A. (2000). SC3 and SC4 hydrophobins have distinct roles in formation of aerial structures in dikaryons of *Schizophyllum commune*. Mol. Microbiol..

[76] Wosten H.A.B. (1994). Interfacial self-assembly of a hydrophobin into an amphipathic protein membrane mediates fungal attachment to hydrophobic surfaces. EMBO J..

[77] Bruns S. (2010). Production of extracellular traps against *Aspergillus fumigatus in vitro* and in infected lung tissue is dependent on invading neutrophils and influenced by hydrophobin RodA. PLoS Pathog..

[78] Paris S. (2003). Conidial hydrophobins of *Aspergillus fumigatus*. Appl. Environ. Microbiol..

[79] Stringer M.A. (1991). Rodletless, a new *Aspergillus* developmental mutant induced by directed gene inactivation. Genes Dev..

[80] Mackay J.P. (2001). The hydrophobin EAS is largely unstructured in solution and functions by forming amyloid-like structures. Structure.

[81] Talbot N.J. (1993). Identification and characterization of *MPG1*, a gene involved in pathogenicity from the rice blast fungus *Magnaporthe grisea*. Plant Cell.

[82] Talbot N.J. (1996). *MPG1* encodes a fungal hydrophobin involved in surface interactions during infection-related development of *Magnaporthe grisea*. Plant Cell.

[83] Askolin S. (2005). The *Trichoderma reesei* hydrophobin genes *hfb1* and *hfb2* have diverse functions in fungal development. FEMS Microbiol. Lett..

[84] Cox A.R. (2007). Surface properties of class II hydrophobins from *Trichoderma reesei* and influence on bubble stability. Langmuir.

[85] Bailey M.J. (2002). Process technological effects of deletion and amplification of hydrophobins I and II in transformants of *Trichoderma reesei*. Appl. Microbiol. Biotechnol..

[86] Humphrey W. (1996). VMD: visual molecular dynamics. J. Mol. Graph..

